# Solar ultraviolet-B exposure and cancer incidence and mortality in the United States, 1993–2002

**DOI:** 10.1186/1471-2407-6-264

**Published:** 2006-11-10

**Authors:** Francis P Boscoe, Maria J Schymura

**Affiliations:** 1 Department of Epidemiology and Biostatistics, School of Public Health, University at Albany, Rensselaer, NY 12144, USA; 2 New York State Cancer Registry, New York State Department of Health, Albany, NY 12237, USA

## Abstract

**Background:**

An inverse relationship between solar ultraviolet-B (UV-B) exposure and non-skin cancer mortality has long been reported. Vitamin D, acquired primarily through exposure to the sun via the skin, is believed to inhibit tumor development and growth and reduce mortality for certain cancers.

**Methods:**

We extend the analysis of this relationship to include cancer incidence as well as mortality, using higher quality and higher resolution data sets than have typically been available. Over three million incident cancer cases between 1998 and 2002 and three million cancer deaths between 1993 and 2002 in the continental United States were regressed against daily satellite-measured solar UV-B levels, adjusting for numerous confounders. Relative risks of reduced solar UV-B exposure were calculated for thirty-two different cancer sites.

**Results:**

For non-Hispanic whites, an inverse relationship between solar UV-B exposure and cancer incidence and mortality was observed for ten sites: bladder, colon, Hodgkin lymphoma, myeloma, other biliary, prostate, rectum, stomach, uterus, and vulva. Weaker evidence of an inverse relationship was observed for six sites: breast, kidney, leukemia, non-Hodgkin lymphoma, pancreas, and small intestine. For three sites, inverse relationships were seen that varied markedly by sex: esophagus (stronger in males than females), gallbladder (stronger in females than males), and thyroid (only seen in females). No association was found for bone and joint, brain, larynx, liver, nasal cavity, ovary, soft tissue, male thyroid, and miscellaneous cancers. A positive association between solar UV-B exposure and cancer mortality and incidence was found for anus, cervix, oral cavity, melanoma, and other non-epithelial skin cancer.

**Conclusion:**

This paper adds to the mounting evidence for the influential role of solar UV-B exposure on cancer, particularly for some of the less-well studied digestive cancers. The relative risks for cancer incidence are similar to those for cancer mortality for most sites. For several sites (breast, colon, rectum, esophagus, other biliary, vulva), the relative risks of mortality are higher, possibly suggesting that the maintenance of adequate vitamin D levels is more critical for limiting tumor progression than for preventing tumor onset. Our findings are generally consistent with the published literature, and include three cancer sites not previously linked with solar UV-B exposure, to our knowledge: leukemia, small intestine, and vulva.

## Background

A wide range of experimental evidence suggests that vitamin D has benefits against a variety of cancer types [1-3]. The primary source of vitamin D for most people in temperate climates, particularly people with light-colored skin, is solar ultraviolet-B exposure [4,5], and the amount of exposure to the sun has been found to correlate inversely with cancer mortality and survival in numerous epidemiological studies. Indeed, this observation has been noted at least since the 1930s [6]. The inverse relationship holds whether long-term cumulative exposure or short-term seasonal exposure is considered [7]. Among the cancer sites for which this inverse relationship has been repeatedly found are prostate [8-16], female breast [8,12,13,15,17-19], and colon and rectum [8,12,13,15,20-23]. Findings have also been reported for ovary [8,13,15,24], uterus [13], bladder [13,15], esophagus [13,15,21], kidney [13,15], lung [13,25], pancreas [13,15,21], stomach [13,15,21], gallbladder and bile duct [15,21], larynx [15], cervix [15], and Hodgkin lymphoma [15,26]. Non-Hodgkin lymphoma has been hypothesized both to be inversely and positively associated with solar UV-B exposure, with the positive hypothesis based on an observed comorbidity with certain skin cancers [27]. Study results exist in support of both hypotheses, though more recent studies favor the inverse association [15,28-33]. For an exhaustive literature review see [2].

Most of the above studies have relied on mortality data exclusively. In this paper, we use both incidence and mortality data, as well as more precise (albeit ecologic) exposure measures and adjustments for confounding variables than has been typical. We calculate age-specific relative risks for incidence and mortality for 32 different cancer sites using data sets of over three million incident cancer cases (1998–2002) and three million cancer deaths (1993–2002) among white non-Hispanics and blacks in the continental United States, after adjusting for socioeconomic, behavioral, occupational, environmental, and geographic risk factors.

## Methods

**Figure 1:**
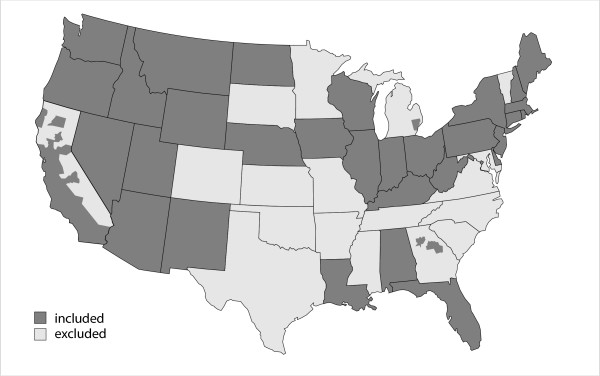
States and counties with available incidence data.

Cancer incidence and mortality were measured at the county level, using incidence data from the North American Association of Central Cancer Registries' CINA Deluxe file [34] and mortality data from the National Cancer Institute's SEER*Stat database [35]. The data consist of approximately 3.1 million incident cancer cases and 3.1 million cancer deaths among white non-Hispanics and 300,000 incident cancer cases and 400,000 cancer deaths among blacks for thirty-two cancer sites (Blacks, with limited sensitivity to geographic variation in solar exposure, serve as a useful comparison group). The included cancer sites were those with at least four thousand incident cases and four thousand deaths, excepting lung cancer as it was used as the basis for adjusting for smoking [36]. Data were stratified by sex, race/ethnicity and ten-year age groups from 35–44 through 85+. For mortality, data were available for 3,108 counties in all states except Hawaii and Alaska, plus the District of Columbia. For incidence, data were available for 1,499 counties in all or parts of 32 states plus the District of Columbia, incorporating about 65% of the United States population (Figure 1). Areas were excluded where county-specific data was unavailable.

Solar UV-B exposure was measured using data from NASA's Total Ozone Mapping Spectrometer (TOMS) [37]. This device has been installed on several spacecraft, including the Earth Probe spacecraft for data from 1996 to 2005. The data consist of an ongoing time series of erythemally-weighted UV-B exposure values for the entire globe, derived from directly-measured noon irradiance values which take into account length of day, cloud conditions, and ozone column. The erythemally-weighted average exposure is the combination of wavelengths from 280–400 nm that best describes the susceptibility of Caucasian skin to sunburn [38]. The shorter wavelengths are the most dangerous, but are blocked by the atmosphere to a greater extent; the result is that in the continental United States about 85% of the contribution to the erythemally weighted average comes from the 300–320 nm range [39]. The wavelengths most important for causing sunburn are similar to those involved in vitamin D production [40].

**Figure 2:**
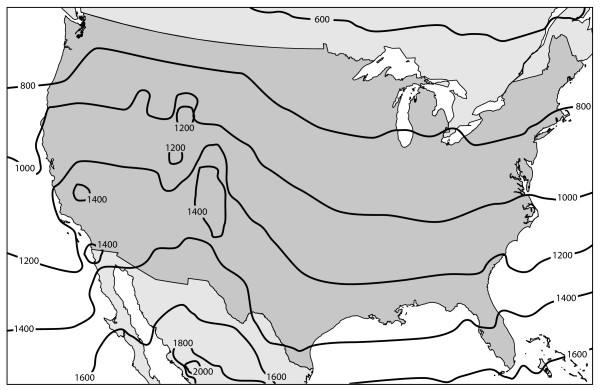
Annual erythemally-weighted ultraviolet-B exposure, kJ/m^2^.

Solar UV-B exposure was based on measurements between September 1, 1996 and August 31, 2003 (seven complete years), at a geographic resolution of one degree. A degree is about 111 kilometers north to south and between 75 and 101 kilometers east to west in the continental United States, depending on latitude. Each measurement location had measurements for between 88% and 97% of the days, with most of the missing values due to the orbital path of the satellite. For each location, we grouped the daily measurements by month in order to calculate monthly averages; the average annual exposure was calculated as the sum of the twelve monthly averages. Exposure values for individual counties were then obtained through areal interpolation using GIS software, resulting in the exposure map seen in Figure 2. As indicated by the figure, exposure correlates closely with latitude, with greater exposure at higher elevations and greater variability in exposure in areas of high relief.

Poisson regression modeling was performed using PROC GENMOD in SAS version 9.1 (SAS Institute, Cary, NC). Poisson regression is a special case of the Generalized Linear Model where the response variable is a count, as is true for cancer cases. The model included ecologic adjustment for the demographic, behavioral and environmental risk factors listed in Table 1. For each cancer site/sex/age/race combination, the relative risk of residing along the northern border of the continental United States (e.g., northern Maine, Minnesota or Washington state) versus the southern border (e.g., southern Florida, Texas or Arizona) was calculated. Specifically, the relative risk corresponding to an exposure of 650 kJ/m^2^-year versus 1540 kJ/m^2^-year was calculated. This is a convenient way of reporting the variation of risk across the continental United States. Predicted relative risks for intermediate locations in the country can be calculated proportionally. For example, the relative risk in North Carolina, Tennessee, and Arkansas, where the exposure is about 1100 kJ/m^2^-year versus the southern border, would be half of that reported for the northern border.

The migration of retirees to the southern United States could be expected to bias results toward the null, because exposure levels there would be overstated. To address this issue, the analysis was repeated after excluding counties with high rates of migration from places with very different solar exposures. The 2000 census county-to-county migration flow file identifies the number of people who moved from and to each county between 1995 and 2000 [41]. Counties were excluded from the analysis if more than one-fifth of the population moved from an area with an average annual exposure that was different by more than 100 kJ/m^2^-year. This resulted in the exclusion of 6 counties in the incidence analysis and 38 counties in the mortality analysis, representing less than 1% of the cases in each analysis. The excluded counties were primarily in the southern and western United States, particularly in Colorado and Florida.

## Results

The relative risks of cancer incidence and mortality for residence along the northern versus southern United States boundary for 32 cancer sites are shown in Tables 2 through 5, grouped into four categories based on their level of association. The categorization took into account magnitude of risk, confidence intervals, consistency between incidence and mortality, and separately calculated age-specific relative risks (not shown). Ten sites showed strong evidence of an inverse association with solar UV-B exposure: bladder, colon, Hodgkin lymphoma, myeloma, other biliary, prostate, rectum, stomach, uterus, and vulva, with two other sites showing this relationship for only one sex (male esophagus, female gallbladder) (Table 2). Weaker evidence of an association was seen for six sites (female breast, kidney, leukemia, non-Hodgkin lymphoma, pancreas, and small intestine), as well as for female esophagus, male gallbladder, and female thyroid (Table 3). No evidence of a relationship was seen for eight sites (bone and joint, brain, larynx, liver, miscellaneous sites, nasal cavity, ovary, soft tissue) as well as male thyroid (Table 4). Solar UV-B exposure was positively associated with five sites of cancer (anus, cervix, melanoma, oral cavity, and other skin) (Table 5).

The largest effects were seen for female gallbladder cancer, with nearly a doubling of risk of both incidence and mortality; uterine cancer, with about a 50% elevated risk; and stomach cancer, with about a 30% elevated risk. Where incidence and mortality risks differed substantially, the higher risk tended to be for mortality, as seen for colon, rectum, other biliary, vulva, breast, esophagus, and miscellaneous sites. One exception was bone and joint cancers, where the risk of incidence is independent of solar UV-B exposure, but the risk of mortality was substantially lower in the south.

For blacks, there was some evidence of association with solar UV-B exposure, but with great inconsistency between sexes and between incidence and mortality for given sites (data not shown). The only site with elevated relative risks for living in the northern versus southern United States that were consistent for both males and females, for both incidence and mortality, was esophagus, with relative risks in the 1.3 to 1.5 range. Evidence of a north-south gradient was also seen for bladder, colon, kidney, larynx, myeloma, and pancreas for female only, and for liver in males only. Female breast cancer was also higher in the north than in the south among blacks, with relative risks of 1.15 (95% confidence interval, 1.11–1.19) for incidence and 1.11 (1.06–1.16) for mortality. While these results overlap those found by Grant in a study focused on blacks [42], it is possible that factors other than vitamin D may be required to explain these differences. Part of the difficulty in interpreting the results for blacks arises from the much smaller number of cases, leading to less certain estimates.

The deletion of cases from high-migration areas had little impact on the results. This remained true even after lowering the migrant population threshold from 20% to 10%, resulting in the exclusion of many more counties (data not shown). Generally, the relative risks of living in the northern versus southern United States were slightly more pronounced when high-migration areas were excluded, as expected. The site with the greatest sensitivity to this variable was gallbladder, which has high mortality rates in the Southwest independent of sun exposure; when some counties in Arizona and New Mexico were excluded, its association with sun exposure increased.

## Discussion

In a recent review article, Giovannucci presents the biological plausibility of the vitamin D hypothesis [1]. Ultraviolet radiation from sunlight produces vitamin D in the skin, which is then hydroxylated in the liver to produce 25(OH)D. Many cell types, including some cancerous cell types, are able to convert 25(OH)D into the more active form of 1,25(OH)_2_D by 1-α-hydroxylase. This function is also performed by the kidneys. Circulating vitamin D activates vitamin D receptors that are located on many cells [43], including cancerous cells, arresting tumor progression and metastasis. The variable efficacy of the vitamin D conversion function by different organs and cell types may account for some of the variation in risk seen between different cancer sites.

For individual cancer sites, the relative incidence risk and relative mortality risk tended to be similar. Among sites with strong evidence of an inverse association with solar UV-B exposure, there was a higher relative mortality risk for breast, colon, rectum, esophagus, other biliary, and vulva. These differences could possibly be related to regional differences in screening, treatment and medical utilization practices; for this reason, incidence data are generally considered preferable to mortality data. But this would imply that there was something about the medical care infrastructure of northern states that was inferior to that of southern states. Recalling that a wide range of factors have been adjusted for in the model, this hypothesis does not make much sense. If anything, the greater concentration of established research hospitals in the older cities in the northern half of the country would be expected to produce an effect opposite the one seen. Even if important regional differences in the medical care infrastructure did exist, there is no reason to expect them to vary latitudinally in the same manner as solar UV-B levels.

A more plausible hypothesis is that the differences between incidence and mortality are related to solar UV-B exposure. Several recent studies that focused on the time of diagnosis and death concluded that vitamin D levels are more relevant to disease progression than disease onset [7,12]. In these studies, little or no pattern was seen in the season of diagnosis (except for a reduction during major holidays, when the level of non-emergency care is reduced), but a strong association was found with the season of death, with death rates higher in winter months when circulating vitamin D levels are at a minimum. Thus it may be that one's overall risk of contracting colon cancer may be moderately influenced by reduced solar UV-B exposure (with an increased risk of 10% to 15% in the northern versus southern United States), while the risk of dying from the disease is more strongly related to reduced solar UV-B exposure (with an increased risk of 25% to 30%).

Incomplete control of confounding may have influenced our findings. For example, there may exist regional variations in viruses and organisms that are believed to be linked with cancer, such as hepatitis B and C infection and aflatoxin for liver cancer, and HPV infection for cervix, vulva, and anal cancer. The enormous racial grouping "white" also may be problematic, insofar as there exist important regional ethnic variations within the grouping as well as geographically variable degrees of racial mixing or miscoding. For example, the high gallbladder cancer mortality rates among whites in the Southwest, which would not be expected under the vitamin D hypothesis, probably reflect the influence of Hispanics and American Indians, who have much higher rates of this disease [44]. Smokeless tobacco use, which is a leading risk factor for oral cancer and strongly concentrated in the rural South, was not adjusted for at all because of insufficient data. Regional variation in diet may have also influenced the findings, particularly for the digestive cancers, although Grant [13] and others argue against this.

Systematic coding problems for several of the cancer sites likely influenced the results. Many advanced cancers metastasize to the bone, so that the geographic pattern of bone and joint cancer mortality, for example, may be influenced by geographically differential misclassification of metastatic tumors as primary tumors. The existence of such misclassification is strongly suggested by sharp differences in rates of miscellaneous cancers between adjacent states [45] (Miscellaneous cancer is a catch-all category incorporating ambiguous, vague or ill-defined sites such as "abdomen" or "thorax", along with unknown sites). Both the tendency to classify cases ambiguously and the tendency to misclassify metastatic tumors are related to resource issues at both the state and hospital levels.

Finally, this being an ecologic study, all of the usual limitations of an ecologic study apply. The ecologic adjustments that were made for smoking, outdoor occupation, particulate matter, and so on were not optimal, relying on proxy measures, survey data, spatial interpolations and other imperfect instruments. The central premise of the study – that where you live determines your sun exposure – while reasonable, is subject to many possible local exceptions, and no data are available that distinguish the solar exposure levels of those with cancer to those without cancer in a given location.

## Conclusion

This paper represents the first effort to relate cancer incidence and solar UV-B exposure on a population basis, to our knowledge. In so doing, we have also corroborated much of the previous research on the relationship between solar UV-B exposure and cancer mortality. We found at least some evidence of an inverse association for nineteen cancer sites and no evidence of an association for eight sites. Five other sites were found to be positively associated with solar UV-B. We are unaware of any previous reports citing leukemia, small intestine, or vulva as being associated with solar UV-B, and only limited (in some cases single) reports identifying bladder, esophagus, gallbladder, Hodgkin's lymphoma, kidney, myeloma, stomach, other biliary, and uterus. We failed to corroborate previous reports linking laryngeal and cervical cancer to reduced solar UV-B exposure; we found no relationship for larynx and found that cervical cancer was positively associated with solar UV-B.

By using both incidence and mortality data, large sample sizes, county-level geographic resolution, high-resolution solar exposure data, and adjustment for numerous confounders, we have attempted to overcome some of the limitations of previous studies of this type. Considered in combination with the ever-growing literature on sunlight, vitamin D and cancer, the evidence is clear that exposure to solar UV-B affords protection against numerous cancers, and that current public health recommendations that advocate little or no sunlight exposure should be revisited – especially since the adverse health effects of vitamin D deficiency are not limited to cancer, but also appear to include type 1 diabetes, multiple sclerosis, rheumatoid arthritis, cardiovascular disease, and osteoporosis [5].

## Competing interests

The author(s) declare that they have no competing interests.

## Authors' contributions

FPB conceived the study design, conducted the literature review, performed the regression analysis, and drafted the manuscript. MJS participated in the study design and selection of statistical methods. Both authors read and approved the final manuscript.

## Pre-publication history

The pre-publication history for this paper can be accessed here:



## Figures and Tables

**Table 1:** Confounding variables adjusted for in the model.

Variable	Definition	Source	Geographic Level	Sex-specific	Race-specific
Age	10-year age groups from 35–44 to 75–84, and 85+	SEER	County	X	X
Poverty	% of households below poverty rate	2000 US Census	County	X	X
Income	Median household income in dollars	2000 US Census	County		X
Smoking	Age-adjusted lung and bronchus cancer mortality rate	SEER	County	X	X
Exercise	% with no exercise in last 30 days	BRFSS 1994–2002 (even years) & 2001	State	X	X
Alcohol	Average number of drinks in past 30 days	BRFSS 1997, 1999, 2001, 2002	State	X	X
Outdoor occupation	% of workers in agriculture, forestry, fishing, hunting, or construction	2000 US Census	County	X	
Urban/rural	% rural population Average annual PM2.5 concentration^a^	2000 US Census	County		
Air quality	(values for ~ 600 counties known, remainder were interpolated)	US EPA AirNow database 2000	County		

^a ^Particulate matter results in an overestimation of surface-level ultraviolet exposure indicated by the TOMS data, since particulate matter absorbs ultraviolet radiation [46]

**Table 2:** Relative risk of incidence and mortality^a ^related to solar UV-B exposure, northern versus southern United States boundary^b^, non-Hispanic whites (95% CI in parentheses): Cancer sites with strongest evidence of an inverse association with solar UV-B exposure.

	Incidence		Mortality	
**Cancer site**	**Males**	**Females**	**Males**	**Females**

Esophagus (males)	1.27 (1.21–1.34)		1.36 (1.31–1.41)	
Stomach	1.42 (1.35–1.49)	1.27 (1.19–1.36)	1.31 (1.26–1.36)	1.26 (1.21–1.32)
Colon	1.11 (1.08–1.13)	1.14 (1.11–1.16)	1.27 (1.24–1.30)	1.24 (1.22–1.27)
Rectum	1.27 (1.23–1.32)	1.14 (1.09–1.18)	1.53 (1.45–1.60)	1.37 (1.30–1.44)
Gallbladder (females)		1.86 (1.66–2.09)		1.98 (1.82–2.16)
Other biliary	1.20 (1.07–1.35)	1.21 (1.07–1.36)	1.56 (1.40–1.75)	1.58 (1.43–1.76)
Uterus		1.49 (1.45–1.53)		1.52 (1.46–1.58)
Vulva		1.18 (1.09–1.29)		1.93 (1.72–2.17)
Prostate	1.20 (1.19–1.22)		1.17 (1.15–1.19)	
Bladder	1.13 (1.10–1.16)	1.15 (1.11–1.20)	1.24 (1.20–1.28)	1.21 (1.15–1.27)
Hodgkin lymphoma	1.16 (1.04–1.29)	1.19 (1.05–1.34)	1.14 (1.00–1.30)	1.25 (1.09–1.43)
Myeloma	1.19 (1.12–1.27)	1.22 (1.14–1.31)	1.16 (1.11–1.22)	1.16 (1.11–1.21)

^a^Adjusted for the variables listed in Table 1 and excluding high-migration counties. Incidence includes states and counties shown in Figure 1 for the period 1998–2002 ; mortality includes entire United States except Alaska and Hawaii for the period 1993–2002.

^b ^Relative risk of receiving annual average of 650 kJ/m^2 ^of erythemally-weighted ultraviolet exposure (a value typical of northern Maine, Minnesota or Washington) versus annual average of 1540 kJ/m^2 ^(a value typical of southern Florida, Texas, or Arizona).

**Table 3:** Relative risk of incidence and mortality related to solar UV-B exposure, northern versus southern United States boundary, non-Hispanic whites (95% CI in parentheses): Cancer sites with weaker evidence of an inverse association with UV-B exposure (see notes for Table 2).

	Incidence		Mortality	
**Cancer site**	**Males**	**Females**	**Males**	**Females**

Esophagus (females)		1.08 (0.99–1.19)		1.21 (1.14–1.28)
Small intestine	1.15 (1.03–1.28)	1.20 (1.07–1.35)	1.07 (0.93–1.22)	1.24 (1.08–1.42)
Gallbladder (males)	1.13 (0.94–1.35)		1.58 (1.38–1.82)	
Pancreas	1.09 (1.05–1.14)	1.17 (1.13–1.22)	1.06 (1.03–1.09)	1.11 (1.08–1.14)
Breast		1.06 (1.05–1.07)		1.15 (1.13–1.17)
Kidney	1.09 (1.05–1.13)	1.17 (1.11–1.22)	1.12 (1.08–1.17)	1.20 (1.14–1.25)
Thyroid (females)		1.10 (1.05–1.16)		1.30 (1.16–1.47)
Non-Hodgkin lymphoma	1.08 (1.05–1.12)	1.09 (1.05–1.12)	1.12 (1.08–1.15)	1.15 (1.12–1.18)
Leukemia	1.09 (1.04–1.13)	1.15 (1.10–1.20)	1.07 (1.03–1.10)	1.09 (1.06–1.13)

**Table 4:** Relative risk of incidence and mortality related to solar UV-B exposure, northern versus southern United States boundary, non-Hispanic whites (95% CI in parentheses): Cancer sites with no evidence of an inverse association with solar UV-B exposure (see notes for Table 2).

	Incidence		Mortality	
**Cancer site**	**Males**	**Females**	**Males**	**Females**

Liver and intrahepatic bile duct	1.01 (0.95–1.08)	1.05 (0.96–1.14)	0.88 (0.85–0.92)	0.85 (0.81–0.89)
Nose, nasal cavity, and inner ear	0.80 (0.68–0.95)	0.85 (0.71–1.03)	0.93 (0.76–1.13)	0.99 (0.80–1.23)
Larynx	0.87 (0.82–0.92)	0.80 (0.72–0.89)	1.04 (0.98–1.11)	0.96 (0.86–1.07)
Bone and joint	0.83 (0.69–1.01)	0.95 (0.78–1.17)	0.62 (0.54–0.72)	0.57 (0.49–0.66)
Soft tissue, including heart	0.84 (0.77–0.92)	0.94 (0.85–1.03)	1.10 (1.02–1.19)	1.23 (1.14–1.33)
Ovary		1.03 (0.99–1.06)		1.09 (1.06–1.11)
Brain and other nervous system	1.08 (1.02–1.14)	1.07 (1.01–1.14)	0.91 (0.87–0.94)	0.86 (0.83–0.90)
Thyroid (males)	1.05 (0.96–1.13)		1.00 (0.87–1.16)	
Miscellaneous sites	0.83 (0.79–0.86)	0.93 (0.89–-0.97)	1.16 (1.13–1.18)	1.23 (1.20–1.26)

**Table 5:** Relative risk of incidence and mortality related to solar UV-B exposure, northern versus southern United States boundary, non-Hispanic whites (95% CI in parentheses): Cancer sites with evidence of an positive association with solar UV-B exposure (see notes for Table 2).

	Incidence		Mortality	
**Cancer site**	**Males**	**Females**	**Males**	**Females**

Oral cavity and pharynx	0.77 (0.74–-0.80)	0.83 (0.79–0.88)	0.79 (0.76–0.83)	0.73 (0.69–0.77)
Anus, anal canal, and anorectum	0.68 (0.59–0.78)	0.71 (0.64–0.79)	0.69 (0.54–0.88)	0.67 (0.56–0.80)
Melanoma	0.72 (0.70–0.74)	0.78 (0.75–0.81)	0.70 (0.67–0.73)	0.83 (0.78–0.87)
Other non-epithelial skin	0.61 (0.55–0.67)	1.04 (0.92–1.18)	0.56 (0.52–0.61)	0.71 (0.63–0.79)
Cervix		0.84 (0.80–0.89)		0.89 (0.84–0.94)
